# Left vs. Right Bundle Branch Block in COVID-19 Patients: Distinct Clinical Presentations and Prognostic Implications

**DOI:** 10.3390/jcm14072310

**Published:** 2025-03-28

**Authors:** Elena Ciurariu, Mara Amalia Balteanu, Marius Georgescu, George Andrei Drăghici, Silviu Gabriel Vlăsceanu, Alina-Florina Șerb, Ramona Cioboată

**Affiliations:** 1 Department of Functional Sciences, Physiology Discipline, Faculty of Medicine, “Victor Babeș” University of Medicine and Pharmacy Timișoara, Eftimie Murgu Square No. 2, 300041 Timişoara, Romania; ciurariu.elena@umft.ro (E.C.); georgescu.marius@umft.ro (M.G.); 2 Department of Pulmonology, Faculty of Medicine, Titu Maiorescu University, 031593 Bucharest, Romania; mara.balteanu@prof.utm.ro; 3 Center of Immuno-Physiology and Biotechnologies (CIFBIOTEH), “Victor Babeș” University of Medicine and Pharmacy Timișoara, Eftimie Murgu Square No. 2, 300041 Timişoara, Romania; 4 Department of Toxicology, Faculty of Pharmacy, “Victor Babeș” University of Medicine and Pharmacy Timișoara, Eftimie Murgu Square No. 2, 300041 Timișoara, Romania; 5 Research Center for Pharmaco-Toxicological Evaluations, Faculty of Pharmacy, “Victor Babeș” University of Medicine and Pharmacy Timișoara, Eftimie Murgu Square No. 2, 300041 Timișoara, Romania; 6 Department of Functional Sciences, Physiology Discipline, Faculty of Medicine, “Carol Davila” University of Medicine and Pharmacy, 050474 Bucharest, Romania; 7 Department of Biochemistry and Pharmacology, Biochemistry Discipline, “Victor Babeș” University of Medicine and Pharmacy Timișoara, Eftimie Murgu Square No. 2, 300041 Timișoara, Romania; aserb@umft.ro; 8 Pneumology Department, University of Medicine and Pharmacy, 200349 Craiova, Romania; ramona_cioboata@yahoo.com

**Keywords:** bundle branch block, COVID-19, cardiac conduction abnormalities, inflammation markers, clinical risk stratification

## Abstract

**Background/Objectives**: COVID-19 is associated with multiple systemic effects, including cardiovascular complications. However, its interplay with cardiac conduction abnormalities remains underexplored. We compared the clinical profile of COVID-19 patients with pre-existing left bundle branch block (LBBB) or right bundle branch block (RBBB) at hospital admission. **Methods**: This study included 100 COVID-19 patients with antecedent BBB (50 LBBB, 50 RBBB). Critical cardiometabolic, renal, hematological, and inflammatory markers were measured. Logistic regression was used to identify key predictors differentiating COVID-19 patients with LBBB and RBBB. Spearman’s correlations were applied to assess intra-strata associations for these variables. **Results**: COVID-19 patients with LBBB patients were significantly more likely to display lower systolic blood pressure (*p* = 0.012) but greater left atrial size (*p* = 0.008), left ventricular diameter (*p* = 0.001), and interventricular septal thickness (*p* = 0.023). Hematological and inflammatory markers differed, with LBBB patients being prone to exhibit higher red cell distribution width (*p* = 0.005), lymphocyte count (*p* < 0.001), neutrophil count (*p* = 0.045), and C-reactive protein (*p* < 0.001). This group also tended to show lower erythrocyte sedimentation rate (*p* = 0.013) and glycated hemoglobin (*p* = 0.045) but higher random glucose (*p* = 0.014). Absolute lymphocyte count, C-reactive protein, and left ventricular diameter were the most robust predictors distinguishing LBBB from RBBB. Significant associations were found exclusively for LBBB, all of them being weak. These predominantly negative relationships indicated an inflammatory origin, and most of them occurred for lymphocyte count. **Conclusions**: COVID-19 patients with LBBB and RBBB present distinct clinical profiles at hospital admission. The former group demonstrates a more adverse baseline clinical profile, particularly in terms of cardiac and inflammatory markers. These findings suggest that pre-existing BBB type may influence disease progression, potentially helping in risk stratification for COVID-19 patients.

## 1. Introduction

Bundle branch block (BBB) is a cardiac conduction disorder associated with delayed (or obstructed) transmission of electrical impulses along the pathways coordinating heartbeats [1]. This imbalance can perturb ventricular contraction, thereby impairing cardiac contractility [1]. Left bundle branch block (LBBB) and right bundle branch block (RBBB) are the two main types of BBB. The former type of BBB is typically linked to pre-existing heart conditions, affecting left ventricular function [2]. RBBB, by contrast, is related to lung diseases and less severe heart conditions (e.g., atrial septal defects, right ventricular dysfunction) [1]. Clinical evidence indicates that LBBB poses greater health risks, resulting in higher mortalities and more advanced cardiac dysfunction [3]. RBBB is more prevalent in older populations [1] and, while serious, is linked to better prognostic outcomes when not accompanied by major cardiac pathologies [3].

The recent coronavirus (COVID-19) pandemic has put a substantial burden on public health systems [4]. Despite primarily targeting the respiratory system, this disease driven by the severe acute respiratory syndrome coronavirus 2 (SARS-CoV-2) can trigger multi-system organ damage due to a “cytokine storm”—an excessive immune response leading to severe inflammation throughout the body [5]. This respiratory illness is associated with multiple cardiovascular conditions, e.g., arrhythmias, acute heart failure, acute coronary syndromes, myocarditis, pericarditis, cor-pulmonale, or pulmonary embolism [6,7]. In particular, older individuals with pre-existing cardiac disorders are at elevated risk for severe, life-threatening SARS-CoV-2 infections and display worse outcomes [7]. However, COVID-19 can contribute to vascular complications in other organ systems besides its direct cardiac effects. For example, this illness is linked to microvascular thrombosis within the retinal vessels [8]. Moreover, COVID-19 patients with exudative macular degeneration—a condition characterized by aberrant subretinal angiogenesis—display worse outcomes compared to the non-exudative form or controls [9].

The aforementioned findings support that COVID-19 functions as a catalyst for the progression of pre-existing heart conditions [10,11]. However, the relationship between this disease and BBB is an area yet to be thoroughly investigated, with the few studies available focusing on COVID-19-associated mortality risks [11,12,13]. It was found that infection with SARS-CoV-2 in the context of pre-existing RBBB is associated with a higher risk of short-term death [12]. Data connecting LBBB and COVID-19 outcomes are somewhat inconsistent. Thus, Zuin et al. reported a higher risk of short-term mortality in subjects with LBBB who contracted COVID-19 [13]. A more recent study by Bannier et al. found no significant association between LBBB and adverse outcomes in these patients, suggesting that additional factors may mediate this relationship [14].

Surprisingly, very little information exists about the clinical profiles of BBB patients with COVID-19 despite their increased risk of cardiac instability, arrhythmias, and hemodynamic compromise [1,2]. Given their heightened cardiovascular vulnerability, understanding their baseline clinical characteristics at the time of hospital admission could provide medical staff with relevant data for early risk stratification and targeted management. Echocardiographic parameters offer crucial insights into cardiac structural and functional changes in the heart [15], playing an important role in detecting COVID-19-induced cardiac alterations [6,7,8]. On the other hand, assessing key organ functions, such as renal, hepatic, and cardiometabolic health, can provide additional data on the systemic impact of COVID-19 in this high-risk population. Renal function plays a pivotal role in determining outcomes in cardiac patients, with even mild impairment increasing the risk of heart failure progression and mortality [16,17]. Moreover, liver function tests and inflammatory markers provide vital information about the systemic effects of COVID-19 [18], reflecting both the level of systemic stress and the body’s inflammatory response to the infection [19].

This study aimed to compare the clinical profile of COVID-19 patients with pre-existing LBBB or RBBB. Our focus on clinical profiles at admission, without follow-up, provides a snapshot of their baseline clinical characteristics rather than disease progression. However, this approach allowed us to systematically control for baseline variables, ensuring a standardized approach to analyzing patient characteristics and facilitating a clear and meaningful comparative analysis. Our findings provide key insights into the multi-organ impact of COVID-19 in patients with pre-existing BBB, serving as a potential basis for future research on this poorly understood interplay.

## 2. Materials and Methods

### 2.1. Design

We conducted a retrospective cross-sectional study to compare the clinical profiles of patients with either LBBB or RBBB who were admitted to the hospital for COVID-19. This pilot exploratory study was performed at the “Victor Babeş” Clinical Hospital of Infectious Diseases and Pneumology in Craiova—here abbreviated as VBCHIDP. Specialized in pneumology and infectious diseases and located in Dolj County (Romania), this regional health facility provides access to a large pool of potentially recruitable patients [20], rendering it an appropriate setting for our research. This single-site investigation was run in compliance with the Declaration of Helsinki (1964) and its later revisions [21,22]. Ethical approval was obtained from the Institutional Ethics Committee (IEB) at the VBCHIDP (approval no. 13111/18.09.2024). Informed consent was obtained and signed by all patients or their respective caregivers. Data were obtained from clinical records of subjects evaluated at VBCHIDP between 10 May 2020 and 1 February 2025.

### 2.2. Protocol

The study population included only COVID-19 patients with documented evidence of pre-existing isolated LBBB or RBBB. The presence of LBBB or RBBB was confirmed based on pre-hospital electrocardiographic (ECG) records, ensuring that these conduction abnormalities were present prior to SARS-CoV-2 infection. This approach reduced the risk of misclassification due to COVID-19-induced conduction disturbances. Moreover, this selection criterion helped mitigate potential confounding effects from acute cardiac complications associated with this respiratory disease.

Other inclusion criteria were adult patients aged 18 years and older, hospital admission for symptomatic COVID-19 with a positive test for SARS-CoV-2 infection via polymerase chain reaction (PCR) testing, and no history of prior pacemaker implantation or other severe structural heart disease unrelated to COVID-19 infection. The main exclusion criteria were incomplete medical records, absence of a confirmatory ECG, presence of other types of conduction abnormalities not classified as isolated LBBB or RBBB (e.g., bifascicular block, complete heart block), significant structural heart disease unrelated to COVID-19 (e.g., severe valvular disease, congenital heart defects), pre-existing severe chronic conditions unrelated to cardiac or COVID-19 status (e.g., advanced malignancies, end-stage renal disease requiring dialysis, or severe liver cirrhosis), and end-of-life or palliative care patients.

COVID-19 severity was not explicitly classified in this study, as we focused on clinical profiles at admission rather than disease progression. The analysis primarily relied on physiological parameters to capture the systemic impact of COVID-19 in LBBB and RBBB patients. However, the need for hospitalization suggests a somewhat moderate disease course.

### 2.3. Data Collection

Data extracted included demographic information (age, sex, and comorbidities), clinical presentation, laboratory results and echocardiographic findings, and outcomes during hospitalization. Data collection focused on key clinical parameters at different organ levels, i.e., cardiometabolic, renal, inflammation, liver, and blood profile markers. Cardiometabolic parameters included a range of indicators related to cardiovascular and metabolic health: (*i*) echocardiographic measurements: left atrial diameter (LAD), left atrial size (LAS), left ventricle diameter (LVD), left ventricular end-systolic volume (LVESV), left ventricular end-diastolic volume (LVEDV), ejection fraction (EF), interventricular septal thickness (IVSd), and pulmonary artery systolic pressure (PASP); (*ii*) blood pressure markers: systolic blood pressure (SBP), diastolic blood pressure (DBP), and heart rate (HR); (*iii*) lipid parameters: total cholesterol, LDL, HDL, and triglycerides; and (*iv*) glycemic markers: random glucose and glycated hemoglobin (HbA1c). Renal health markers included serum creatinine, serum urea, serum uric acid, serum sodium, and serum potassium. Liver function was assessed via alanine aminotransferase (ALT) and aspartate aminotransferase (AST). Hematological markers analyzed included hemoglobin, red cell distribution (RDW-CV, RDW-SD), absolute lymphocyte count (ALC), absolute neutrophil count (ANC), and routinely used inflammation metrics, i.e., erythrocyte sedimentation ratio (ESR), and C-reactive protein (CRP). COVID-19 severity was not explicitly classified in this study, as the focus was on clinical profiles at hospital admission rather than disease progression. However, all patients required hospitalization, indicating at least a moderate disease course. The analysis primarily relied on physiological parameters to capture the systemic impact of COVID-19 in LBBB and RBBB patients.

### 2.4. Statistical Analysis

Statistical analysis was run with Statistica version 10 software (StatSoft Inc., Tulsa, OK, USA). Categorical data related to gender, origin, presence of diabetes, and smoking status are provided as frequencies with the corresponding percentages. Continuous variables are expressed as median with lower quartiles and upper quartiles [23]. A Mann–Whitney U-test was conducted to assess intergroup homogeneity concerning age [23]. Comparisons between LBBB and RBBB groups for categorical variables were conducted using chi-square tests with Yates’s correction for continuity [24]. Logistic regression was used to identify the best predictors separating COVID-19 patients with LBBB and RBBB. This approach enabled us to determine the independent effect of each predictor while controlling for the influence of other variables in the model [25]. Type 3 Likelihood Ratio (LR) tests were used, which inherently focus on the strongest predictors and mitigate the issue of excessive comparisons. A chi-square threshold of 10—corresponding to a *p*-value of ≈ 0.0016—was chosen to ensure the identification of the most influential variables. Such a criterion minimizes the inclusion of weak predictors, simplifies the model for interpretation, and aligns with domain-specific requirements for practical significance [25]. For each stratum, Spearman’s correlations were then conducted for these predictors and the other continuous variables [26]. Statistical significance was defined as a two-sided *p*-value less than 0.05 [27].

## 3. Results

### 3.1. Health Profiles of COVID-19 Patients with LBBB and RBBB

After matching for age and sex, data from 50 COVID-19 patients with LBBB and 50 with RBBB were collected for this pilot investigation. Their sociodemographic characteristics are provided in Table 1. There were no significant intergroup differences in sex distribution, area of origin, glycemic profile, and smoking status (Table 1). Table 2 shows the median value (with lower quartiles and upper quartiles) for the continuous parameters investigated. COVID-19 patients with LBBB and RBBB were of similar age (Mann–Whitney, *p* = 0.349). Both groups showed elevated SBP and PSAP but below-normal median EF values (Table 2). The other cardiac parameters were within (close to) the physiological range (Table 2). The most noticeable inter-strata differences were lower SBP, EF, and PSAP but higher LVD in LBBB patients with COVID-19.

The median values for hematocyte-related parameters (RDW-CV, RDW-SD) and the other blood parameters (hemoglobin, lymphocytes, neutrophils) were within the physiological range (Table 2). In contrast, inflammation markers (ESR, CRP) were at the upper level of normal range or slightly elevated (Table 2). Although the random glucose was within the normal limits, median HbA1c was mildly elevated in both strata (Table 2). With respect to renal profile, serum urea levels were in the upper normal range in LBBB patients and slightly increased in RBBB patients (Table 2). Almost all patients were hyperuricemic despite showing normal values for serum sodium and serum potassium (Table 2). Lipid metrics and liver parameters were also within the physiological range (Table 2).

Table 3 provides the key parameters of the Type 3 (LR) test. Systolic blood pressure was a significant determinant of bundle branch block type; that is, COVID-19 patients with lower SBD were more likely to have LBBB (Table 3). In contrast, elevated LAS, LVD, and IVSd were all linked to higher odds of having LBBB (Table 3). The other cardiac parameters were not significantly associated with the type of conduction abnormality (Table 3).

Among hematological parameters, RDW-SD, ALC, ANC, CRP, and ESR emerged as significant separators between LBBB and RBBB individuals with COVID-19 (Table 3)**.** For the first four metrics, high values were associated with greater odds of having LBBB. An inverse association was observed for the latter parameter (Table 3). The parameters of glycemic control also served as significant differentiators between LBBB and RBBB in the context of COVID-19 (Table 3). Individuals with COVID-19 and elevated HbA1c were less likely to have LBBB (Table 3). Elevated random glucose, on the other hand, was associated with a greater probability of having LBBB, but its effect was very weak (Table 3). However, renal, lipid, and liver indicators did not account for the separation between LBBB and RBBB in patients with COVID-19 (Table 3).

The most robust predictor of bundle branch block type in the context of COVID-19 was ALC, followed by CRP and LVD, with each variable showing chi-square values well above 10 (Table 3). RDW-SD, SBP, ESR, random glucose, IVSd, and HbA1c had moderate importance in delineating LBBB from RBBB. On the other hand, LAS and ANC were minor predictors of bundle branch block type despite reaching statistical significance.

### 3.2. Correlational Patterns of LBBB and RBBB in the Context of COVID-19

Intergroup correlational patterns were analyzed for LVD, ALC, and CRP—the most influential predictors for differentiating LBBB and RBBB in the context of COVID-19. The magnitude of these associations is provided in Table 4. We identified significant associations exclusively for LBBB, all of which were weak and predominantly negative (Table 4). LVD was inversely correlated with DBP (Table 4). Similar relationships were identified between ALC and RDW-SD, ESR, and HDL, as well as between CRP and HR (Table 4). In contrast, ALC correlated directly with serum uric acid (Table 4).

## 4. Discussions

This is one of the few studies examining the interplay between pre-existing heart conduction abnormalities and COVID-19. While previous research focused on COVID-19 outcomes in patients with LBBB and RBBB [12,13,14], we compare their clinical profiles at hospital admission. This approach could help establish an initial framework for improved risk stratification for these subjects at high risk for cardiac complications.

Besides comparable distributions for sex, area of origin, diabetes status, and smoking status, the two strata were of a similar age. Any observed differences are therefore attributable to the conduction abnormality (LBBB vs. RBBB) rather than to age differences. Systolic blood pressure, pulmonary artery systolic pressure, and ejection fraction were the only cardiac parameters outside the normal range. Indeed, cardiac conduction disorders can indeed alter cardiac hemodynamics, but this interplay is complex and multifaceted. Chronic hypertension promotes myocardial stiffness and left ventricular hypertrophy, eventually progressing to LBBB and leading over time to a progressive decline in ejection fraction [2,3,28]. On the other hand, high systolic blood pressure may increase left-sided filling pressures. In turn, severe pulmonary hypertension and right ventricular pressure overload can contribute to the development of RBBB [1,29,30,31].

No study to date has conducted a direct comparative analysis of blood pressure in patients with LBBB or RBBB. Here, COVID-19 patients with lower systolic blood pressure were more likely to have LBBB. Research findings indicate that LBBB can be associated with hypotension [32,33,34,35]; however, no comparable data are available for RBBB. It is plausible that lower systolic blood pressure seen in LBBB derives from reduced cardiac output caused by this conduction abnormality [36]. In contrast, COVID-19 generally does not directly reduce cardiac output to the extent that LBBB does [37].

Elevated left atrial size, left ventricular diameter, and interventricular septal thickness were linked to a higher likelihood of having LBBB. Left atrial enlargement indirectly reflects the effect of high blood pressure in the left ventricle [38]. Increased left ventricular diameter is a hallmark of left ventricular dilation [39]. Interventricular septal thickness (IVST) is also closely related to left ventricular hypertrophy [40]. These structural remodeling events on the left side of the heart serve as primary drivers for LBBB development. The greater hemodynamic burden of the left ventricle—characterized by enhanced afterload sensitivity, greater wall stress, and prolonged depolarization—renders LBBB more vulnerable to conduction delays. Furthermore, the anatomical complexity and deeper location of the left bundle branch fibers make them susceptible to pressure-related or ischemic damage, especially in conditions affecting left ventricular function [38,39]. Given these mechanisms, LBBB is far more likely than RBBB in conditions involving left-sided structural changes.

Left ventricular diameter was the most influential cardiac parameter distinguishing the two strata. LBBB typically results from degenerative conduction system disease, left ventricular myocardial pathology, or conditions leading to severe left ventricular hypertrophy. Given the early branching and extensive distribution of the left bundle within the left ventricle, LBBB often indicates widespread cardiac disease [40]. Importantly, this metric showed minimal associations with the other variables analyzed. Left ventricular diameter is hence shaped by intrinsic cardiac remodeling rather than broader systemic conditions (e.g., metabolic imbalances, inflammatory responses). The weak but significant inverse correlation between this parameter and diastolic blood pressure could be attributed to left ventricle dysfunction and remodeling in LBBB, leading to lower diastolic blood pressure [41]. This association may also result from impaired ventricular–arterial coupling in LBBB, which reduces vascular resistance, or from neurohormonal activation affecting systemic blood pressure regulation [42,43,44].

Most hematological parameters distinguished LBBB from RBBB. RDW-SD, absolute lymphocyte count, and absolute neutrophil count—routinely measured in a complete blood count test—are commonly associated with systemic inflammation, oxidative stress, or underlying comorbidities. All these markers are clinically relevant indicators of immune function and prognosis in various diseases, including COVID-19 and cardiac conditions [45,46]. However, these physiological variables are best interpreted in conjunction with other clinically significant inflammatory markers—in particular, C-reactive protein or erythrocyte sedimentation rate—to provide an accurate assessment of patient health [47]. These indicators showed here values at the upper end or above the normal range, suggesting the existence of an ongoing inflammatory response. With a half-life of 18–20 h, the C-reactive protein is the most useful marker of acute systemic inflammation, including infections, sepsis, COVID-19, trauma, and myocardial infarction [48]. With a much longer half-life (days to weeks), the erythrocyte sedimentation rate, on the other hand, is more sensitive and specific to chronic inflammation, being useful for monitoring autoimmune diseases, chronic infections, and inflammatory disorders [49].

The aforementioned parameters tended to be significantly elevated in LBBB versus RBBB. These findings lent support to a more severe and localized inflammatory response in LBBB and a longer-term, persistent inflammatory pattern in RBBB. Indeed, COVID-19 triggers a hyperinflammatory response—also known as a cytokine storm—which is marked by elevated levels of pro-inflammatory cytokines such as IL-6, TNF-α, and IL-1β [50]. Associated with a hypercoagulable state, this inflammatory milieu drives myocardial inflammation and damage and potentially to new-onset LBBB [51]. If the inflammatory response was purely due to COVID-19, comparable elevations would be expected in both groups. The observed inflammatory differences are, hence, multifactorial, potentially related to the cardiac pathophysiology inherent to each conduction abnormality.

Interestingly, lymphocyte count was the most influential predictor. A retrospective cohort review by Wagner et al. found that low lymphocyte levels yield a worse prognosis in COVID-19 patients admitted to the Intensive Care Unit [52]. Several studies also demonstrated that a higher neutrophil-to-lymphocyte ratio (NLR) on admission is associated with poor outcomes in COVID-19 patients [53,54]. From this point of view, a higher lymphocyte count in LBBB suggests a less severe immune response or better immune resilience compared to RBBB. While a direct link between lymphocyte count and BBB is yet to be reported, its existence cannot be excluded. In fact, the role of lymphocytes in inflammatory cardiac conditions suggests a potential pathway for conduction abnormalities. For example, decreased lymphocyte count is connected to poorer outcomes in patients with heart failure, chronic ischemic heart disease, and acute coronary syndromes [55].

Random glucose and HbA1c showed different patterns, although both serve as critical indicators of glycemic control [56]. The markedly greater impact observed in the case of HbA1c most likely reflects its role as a hallmark of long-term glycemic control [57]. The lower likelihood of COVID-19 patients with elevated HbA1c to have LBBB aligns with existing data. Following an extensive review of previous studies, Mohaved identified a higher prevalence of RBBB in diabetes mellitus but no association with LBBB [55].

COVID-19 patients with LBBB showed weak but significant correlations between key distinguishing variables and biomarkers of inflammation, lipid metabolism, and cardiac function. It is well documented that cytokine storms can cause lymphocyte levels to decline and erythrocyte sedimentation rate to rise in SARS-CoV-2 infection, most likely reflecting an intensified inflammatory response [58,59]. This is in line with the negative correlation observed here between the lymphocyte count and both RDW-SD and erythrocyte sedimentation rate. In this context, lymphopenia may hence signal immune exhaustion, whereas an increased erythrocyte sedimentation rate may indicate persistent inflammation and hypercoagulability. The inverse association between lymphocyte count and HDL, alongside its positive correlation with serum uric acid, further supports inflammation as a key driver of these relationships. HDL exerts potent anti-inflammatory and cardioprotective effects, with lower HDL levels in COVID-19 patients yielding worse outcomes [60,61]. Elevated uric acid is typically regarded as a marker of oxidative stress and metabolic dysfunction—major contributors to endothelial and cardiac conduction abnormalities. However, this compound is central to stress resilience, with both low and high levels being connected to the worst outcomes in COVID-19 patients [62]. Finally, inflammation may account for the inverse association seen here between the C-reactive protein and heart rate. The available data provide a degree of support for this assumption, with COVID-19 severity correlating directly with C-reactive protein concentrations [63] but inversely with heart rate variability [64]. These findings underscore the potential utility of these variables in understanding the distinct pathophysiology of LBBB in the context of COVID-19, whereas RBBB displayed a lack of significant intra-strata correlations. Nonetheless, given potential confounding factors (e.g., pre-existing conditions, treatment variations, demographic differences), measurement variability, and heterogeneity of COVID-19 patients, these relationships warrant careful interpretation.

According to the aforementioned findings, LBBB patients presenting to the hospital with COVID-19 tend to display lower systolic blood pressure but larger left atrial size, left ventricle diameter, and interventricular septal thickness than those with RBBB. These clinical features lend support to a more advanced stage of cardiac remodeling and dysfunction compared to their counterparts. This altered cardiac function overlapped with higher C-reactive protein and leukocyte counts and significant, inflammation-related correlational patterns, indicating a more vigorous—and potentially damaging—acute inflammatory response. This pattern is often associated with a higher risk of complications, such as cytokine storms or secondary infections [65,66]. The combined burden of these factors suggests that individuals with LBBB display a more adverse clinical profile at hospital admission with COVID-19 than subjects with RBBB

This study is subject to several limitations. All data were collected at a single time point and from one clinical site. Temporal or causal relationships between predictors and outcomes cannot be hence established. These findings may also not be generalizable to broader, more diverse patient populations. However, this design is typical in pilot studies, concentrating on a specific, well-defined patient group and controlling for key initial variables to provide a solid baseline for understanding patient profiles [67].

We did not include COVID-19 severity in our analysis. Several factors influenced our decision. First, this was not feasible considering the retrospective nature of the study and potential inconsistencies in severity grading across different medical records [68]. Second, incorporating severity data for a single time point (admission) could lead to misclassification and confounding since COVID-19 severity can evolve over time [7,19], being influenced by multiple in-hospital interventions [18]. Third, our study aimed to help in understanding potential cardiovascular implications at disease onset [10,11], which can be relevant for early risk stratification. Understanding the initial clinical characteristics at admission, on the other hand, is critical for immediate risk assessment and management, irrespective of evolving disease severity.

The design of the study did not allow us to separate the effects of SARS-CoV-2 infection and BBB. This makes it challenging to determine their precise contribution to the clinical panel of patients investigated. Nonetheless, the medical literature yields relevant data to derive pertinent assumptions. That is, changes in markers of systemic inflammation (PCR, VSH, RDW-SD) are more likely related to COVID-19 [47,48,49,50,58,59,63], while cardiac parameters to conduction problems (LBBB or RBBB) [1,2,3,38,39,40]. Whether these changes directly influence conduction disturbances or merely reflect broader systemic changes remains an open question requiring mechanistic studies.

Treatment details were not provided in this study as our aim was to compare the baseline clinical characteristics of LBBB and RBBB patients hospitalized with COVID-19. However, standard management protocols at the study site included antiviral therapy (e.g., remdesivir), corticosteroids for severe cases, and anticoagulation when indicated. While treatments may have influenced some laboratory parameters, their effect on the fundamental differences observed between the LBBB and RBBB groups is likely minimal. Moreover, the exclusion of treatment details is a common approach in pilot retrospective studies since these studies primarily focus on preliminary data exploration, hypothesis generation, and identifying trends rather than conducting a comprehensive assessment of therapeutic interventions [68].

Correlation analysis yielded mostly weak associations, yet many inflammatory and cardiac parameters were consistently altered in LBBB vs. RBBB patients, suggesting a systemic effect rather than random associations. This consistency across multiple biomarkers reinforces the hypothesis that conduction abnormalities may be associated with broader physiological disturbances rather than isolated variations. The clustering of altered parameters further supports the notion that these changes reflect underlying pathophysiological mechanisms rather than spurious findings. As a result, while each correlation on its own may be weak, their collective presence and alignment with known pathophysiological patterns enhance their interpretability and clinical relevance.

Finally, this study lacks longitudinal follow-up. This drawback limits insights into disease progression, long-term outcomes, or the dynamic interplay between cardiac parameters and COVID-19 evolution. A comparative analysis of COVID-19 outcomes in LBBB vs. RBBB is especially noteworthy since empirical data on this topic are scarce and not entirely consistent [12,13,14]. On the other hand, further investigation is warranted to determine whether the inflammatory differences observed are primarily a consequence of COVID-19 progression or reflect pre-existing cardiovascular inflammation. Prospective studies incorporating repeated assessments of inflammatory and cardiac markers are also needed because they could help disentangle the specific contributions of SARS-CoV-2 infection and pre-existing conduction abnormalities to patient outcomes. Larger cohort studies with robust stratification based on conduction disturbances may provide more conclusive insights into differential risk profiles associated with LBBB and RBBB in the context of COVID-19. Future research may also benefit from integrating advanced computational models, such as machine learning-based risk prediction. For example, HeartEnsembleNet—an innovative hybrid ensemble learning framework—has demonstrated potential in refining cardiovascular risk assessment by leveraging multi-source clinical data to enhance predictive accuracy [69]. Such methodologies could complement traditional logistic regression approaches, potentially improving personalized risk assessment in patients with pre-existing conduction abnormalities.

In addition, recent large-scale studies have explored cardiovascular complications in diverse settings. Thus, a nationwide 8-year follow-up study in South Korea examined long-term cardiovascular risks in patients undergoing spinal fusion surgery [70]. While unrelated to conduction abnormalities per se, this research underscores the need for long-term surveillance of cardiovascular complications across different patient populations. Similar longitudinal approaches could provide valuable insights into the progression of LBBB and RBBB in the context of COVID-19 and explore whether conduction disturbances influence COVID-19-related complications beyond their direct impact on cardiac function. Emerging evidence suggests that pre-existing cardiovascular conditions may exacerbate the severity of viral infections, yet the interplay between conduction abnormalities and COVID-19 remains insufficiently understood. Future investigations should therefore integrate electrophysiological assessments with comprehensive inflammatory profiling to elucidate potential mechanistic links.

## 5. Conclusions

This is the first study to compare clinical profiles of COVID-19 patients with pre-existing LBBB and RBBB at hospital admission. LBBB patients were prone to exhibit lower systolic blood pressure but increased left atrial size, larger left ventricular diameter, and greater interventricular septal thickness—suggestive of advanced maladaptive cardiac remodeling and dysfunction. Their inflammatory profile was also more pronounced, with heightened C-reactive protein levels, high variability of erythrocyte size, and elevated leukocyte count, alongside significant correlations between inflammation-related biomarkers. However, these patients tended to display better long-term glycemic control. Leukocyte count, CRP, and left ventricular diameter were the most robust predictors of conduction abnormality type. These findings indicate that LBBB patients presenting at the hospital with COVID-19 show a more detrimental clinical phenotype compared to their RBBB counterparts, especially with regard to cardiac and inflammatory markers. However, further research is required to explore the potential long-term implications of these findings.

## Figures and Tables

**Table 1:** Sociodemographic characteristics in COVID-19 patients with LBBB and RBBB.

Characteristic	Strata	LBBB	RBBB	*p*
Sex	Male	28 (56%)	23 (46%)	0.424
	Female	22 (44%)	27 (54%)	
Origin	Rural	30 (60%)	21 (42%)	0.110
	Urban	20 (40%)	29 (58%)	
Diabetes	Yes	18 (36%)	25 (50%)	0.225
	No	32 (64%)	25 (50%)	
Smoking	Yes	37 (74%)	31 (62%)	0.284
	No	13 (26%)	19 (38%)	

Data are shown as absolute values with the corresponding percentages in parentheses. Marked values (*) show significant differences compared to LBBB (chi-square tests with Yates’s correction, ***—*p* < 0.001, **—*p* < 0.01, *—*p* < 0.05).

**Table 2:** Measured values for selected parameters in COVID-19 patients with LBBB and RBBB.

Characteristic	LBBB	RBBB	Reference Range
Age (years)	70 (63; 71)	71 (66; 79)	
SBP (mm Hg)	140 (120; 136)	150 (140; 160)	90–130
DBP (mm Hg)	80 (75; 90)	80 (79; 91)	60–80
HR (bpm)	88 (80; 108)	90 (79; 110)	60–100
EF (%)	40 (30; 50)	45 (40; 55)	50–70
LAS (mm)	42 (38; 48)	43 (38; 46)	<41
LAD (mm)	49.5 (44; 57)	49.5 (43; 52)	25–53
LVD (mm)	53 (48; 58)	49 (45; 53)	39–59
IVSd (cm)	1.2 (1; 1.3)	1.1 (1; 1.2)	0.6–1.2
PSAP (mm Hg)	47 (37; 65)	52 (45; 65)	<40
RDW-CV (%)	15.10 (14.3; 16.1)	14.5 (13.6; 16.4)	11.5–15.4
RDW-SD (fL)	46.7 (44.6; 50.2)	44.9 (43.6; 50.2)	39–46
Hemoglobin (g/dL)	13.1 (11.8; 14.2)	13 (11.6; 14.0)	12.1–17.2
ALC (cells/µL)	1715 (1120; 2260)	1290 (810; 1820)	1000–4800
ANC (cells/µL)	6175 (4410; 7620)	5070 (2970; 7140)	2500–7000
ESR (mm/h)	30 (18; 48)	33 (18; 42)	0–30
CRP (mg/L)	14 (9; 22)	10 (3; 20)	<10
Random glucose (mg/dL)	129 (112; 165)	133 (106; 174)	<200
HbA1c (%)	6.6 (6; 7,8)	6.85 (6.1; 7.9)	<6.5
Serum urea (mg/dL)	47.15 (41; 69)	54 (31; 65)	<49
Serum uric acid (mg/dL)	7.8 (6.9; 10.3)	9.1 (7; 10.4)	3–7
Serum creatinine (mg/dL)	1.09 (0.69; 1.35)	0.95 (0.79; 1.50)	0.6–1.3
Serum sodium (mmol/L)	140 (137; 142)	141 (137; 142)	135–147
Serum potassium (mmol/L)	4.3 (4; 4.7)	4.15 (4; 4.3)	3.6–5.2
Total cholesterol (mg/dL)	150 (116; 181)	153 (113; 187)	<200
LDL (mg/dL)	92 (72; 134)	103 (80; 130)	<130
HDL (mg/dL)	41 (35; 49)	39 (33; 49)	>40
Triglycerides (mg/dL)	103 (88; 136)	118 (83; 159)	<150
AST (UI/L)	29 (21; 42)	24 (19; 36)	5–56
ALT (UI/L)	27 (18; 39)	20 (17; 24)	9–40

Left bundle branch block, LBBB; right bundle branch block, RBBB. The data are expressed as median values, with the corresponding lower and upper quartiles being provided in parentheses.

**Table 3:** Predictors of LBBB vs. RBBB: logistic regression findings.

Variable	Estimate	Chi-square	*p*
SBP	−0.240	6.29	0.012 *
DBP	0.139	0.88	0.347
HR	0.004	0.03	0.872
EF	0.107	0.52	0.471
LAS	0.549	1.98	0.008 **
LAD	−0.216	2.25	0.133
LVD	0.790	11.16	0.001 **
IVSd	28.972	5.15	0.023 *
PSAP	−0.039	0.28	0.596
RDW-CV	0.580	1.41	0.236
RDW-SD	0.591	7.77	0.005 **
Hemoglobin	0.812	1.81	0.179
ALC	2.349	22.06	<0.001 ***
ANC	0.001	3.86	0.049 *
ESR	−0.162	6.14	0.013 *
CRP	0.464	13.57	<0.001 ***
Random glucose	0.078	6.02	0.014 **
HbA1c	−1.137	4.02	0.045 *
Serum urea	−0.039	0.48	0.489
Serum uric acid	−0.549	2.19	0.139
Serum creatinine	7.496	2.93	0.087
Serum sodium	0.225	1.17	0.280
Serum potassium	3.476	1.76	0.185
Total cholesterol	−0.014	0.14	0.713
LDL	0.148	2.86	0.091
HDL	0.026	0.35	0.552
Triglyceride	−0.010	0.22	0.642
AST	−0.092	1.79	0.181
ALT	0.115	3.36	0.067

Marked values (*) indicate significant differences versus COVID-19 patients with RBBB (Type 3 LR test, ***—*p* < 0.001, **—*p* < 0.01, *—*p* < 0.05).

**Table 4:** Inter-strata correlational analysis.

	LBBB			RBBB		
	LVD	ALC	CRP	LVD	ALC	CRP
SBP	0.02	−0.11	0.07	0.17	0.13	−0.03
DBP	−0.26 *	−0.06	−0.05	−0.03	0.12	0.27
HR	−0.11	0.01	−0.24 *	−0.03	0.03	0.53
EF	0.06	−0.02	−0.01	−0.33	0.14	0.14
LAS	0.12	−0.12	−0.02	−0.03	0.34	−0.24
LAD	0.05	−0.02	−0.21	0.15	0.31	−0.19
LVD	1.00	−0.09	−0.04	1.00	0.26	−0.09
IVSd	0.02	−0.02	0.03	−0.03	0.15	0.18
PSAP	0.15	−0.09	−0.19	−0.19	0.03	0.15
RDW-CV	−0.06	−0.21	−0.08	−0.02	−0.08	0.33
RDW-SD	0.01	−0.39 *	−0.04	0.22	0.11	−0.03
Hemoglobin	−0.05	0.21	−0.08	0.04	0.10	−0.32
ALC	−0.09	1.00	0.27	0.15	1.00	0.07
ANC	0.04	0.08	−0.10	0.10	0.04	−0.34
ESR	0.04	−0.23 *	0.21	0.02	0.07	0.03
CRP	−0.04	0.21	1.00	0.12	−0.21	1.00
Random glucose	−0.05	−0.05	−0.02	−0.34	−0.21	0.11
HbA1c	0.11	0.20	0.21	−0.07	−0.12	−0.02
Serum urea	0.18	−0.13	0.06	−0.14	−0.11	0.27
Serum uric acid	−0.06	0.31 **	−0.03	0.06	−0.38	0.21
Serum creatinine	0.11	0.05	0.14	0.02	−0.05	0.18
Serum sodium	−0.04	0.02	−0.15	−0.02	−0.06	0.05
Serum potassium	0.05	−0.05	−0.03	−0.22	−0.17	0.23
Total cholesterol	−0.11	0.09	−0.04	0.15	−015	−0.15
LDL	−0.07	0.10	−0.05	−0.12	0.12	−0.02
HDL	0.09	−0.29 *	0.03	0.12	−0.31	−0.08
Triglyceride	−0.18	0.03	−0.10	0.08	0.17	−0.25
AST	−0.11	0.04	−0.07	−0.02	−0.15	028
ALT	−0.21	−0.05	−0.15	−0.12	−0.12	0.28

Data are shown as the absolute values of Spearman’s correlations. Marked bolded values (*) indicate significant associations (Spearman’s correlations, ***—*p* < 0.001, **—*p* < 0.01, *—*p* < 0.05).

## Data Availability

All the data generated or analyzed during this study are included in this published article.
